# Crystal Structure, Steady-State, and Pre-Steady-State Kinetics of *Acinetobacter baumannii* ATP Phosphoribosyltransferase

**DOI:** 10.1021/acs.biochem.3c00551

**Published:** 2023-12-27

**Authors:** Benjamin J. Read, Andrew F. Cadzow, Magnus S. Alphey, John B. O. Mitchell, Rafael G. da Silva

**Affiliations:** † School of Biology, Biomedical Sciences Research Complex, University of St Andrews, St Andrews, KY16 9ST, United Kingdom; ‡ EaStCHEM School of Chemistry, Biomedical Sciences Research Complex, University of St Andrews, St Andrews, KY16 9ST, United Kingdom

## Abstract

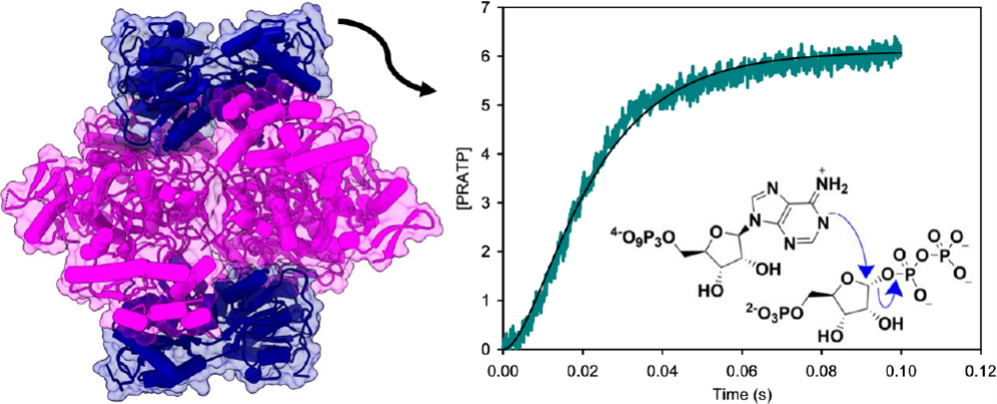

The first step of histidine biosynthesis in *Acinetobacter baumannii*, the condensation of ATP and 5-phospho-α-d-ribosyl-1-pyrophosphate to produce *N*^1^-(5-phospho-β-d-ribosyl)-ATP (PRATP) and pyrophosphate, is catalyzed by the hetero-octameric enzyme ATP phosphoribosyltransferase, a promising target for antibiotic design. The catalytic subunit, HisG_S_, is allosterically activated upon binding of the regulatory subunit, HisZ, to form the hetero-octameric holoenzyme (ATPPRT), leading to a large increase in *k*_cat_. Here, we present the crystal structure of ATPPRT, along with kinetic investigations of the rate-limiting steps governing catalysis in the nonactivated (HisG_S_) and activated (ATPPRT) forms of the enzyme. A pH-rate profile showed that maximum catalysis is achieved above pH 8.0. Surprisingly, at 25 °C, *k*_cat_ is higher when ADP replaces ATP as substrate for ATPPRT but not for HisG_S_. The HisG_S_-catalyzed reaction is limited by the chemical step, as suggested by the enhancement of *k*_cat_ when Mg^2+^ was replaced by Mn^2+^, and by the lack of a pre-steady-state burst of product formation. Conversely, the ATPPRT-catalyzed reaction rate is determined by PRATP diffusion from the active site, as gleaned from a substantial solvent viscosity effect. A burst of product formation could be inferred from pre-steady-state kinetics, but the first turnover was too fast to be directly observed. Lowering the temperature to 5 °C allowed observation of the PRATP formation burst by ATPPRT. At this temperature, the single-turnover rate constant was significantly higher than *k*_cat_, providing additional evidence for a step after chemistry limiting catalysis by ATPPRT. This demonstrates allosteric activation by HisZ accelerates the chemical step.

## Introduction

*Acinetobacter baumannii* is a Gram-negative bacillus belonging to the Moraxellaceae family, capable of causing life-threatening nosocomial infections in the lung, bloodstream, and urinary tract.^1,2^ Carbapenem-resistant *A. baumannii* is resistant to a multitude of antibiotics and was ranked as the top priority in the World Health Organization’s list of drug-resistant bacteria against which novel antibiotics are needed.^3^ Ventilator-associated pneumonia is one of the most prevalent manifestations of *A. baumannii* infection, and when caused by multidrug-resistant strains of the pathogen, it is associated with high mortality rates.^2,4,5^ The development of novel antibiotics against *A. baumannii* is of paramount importance,^1,3^ and the characterization of promising molecular targets can help inform drug design.^6^

**Scheme 1:**
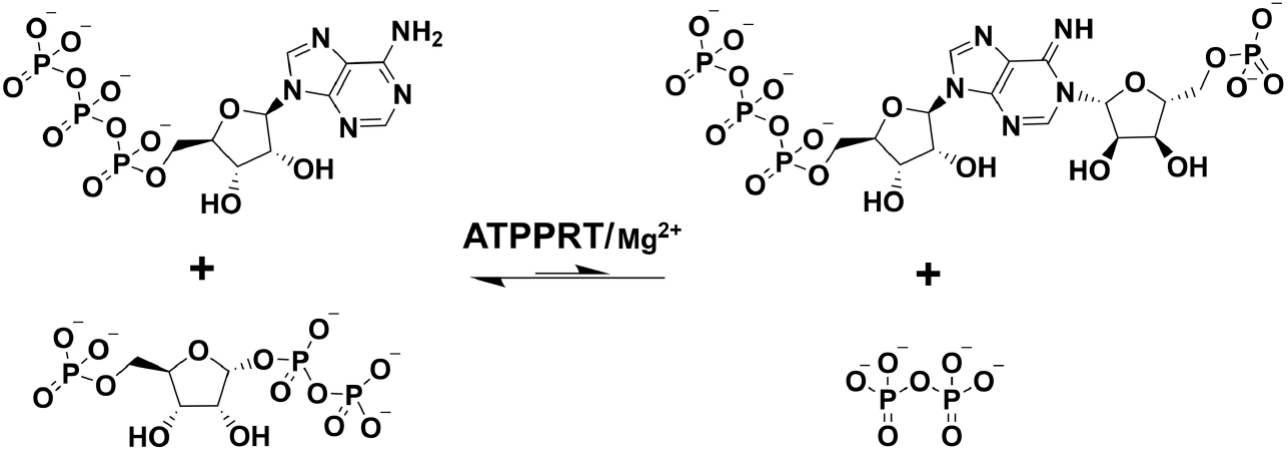
ATPPRT-Catalyzed Nucleophilic Substitution Reaction

The histidine biosynthesis pathway is necessary for *A. baumannii* to persist in the lungs and establish pneumonia, presenting opportunities for the design of novel drugs against this pathogen.^7,8^ The first step of histidine biosynthesis is the reversible and Mg^2+^-dependent reaction of ATP and 5-phospho-α-d-ribosyl-1-pyrophosphate (PRPP) to generate *N*^1^-(5-phospho-β-d-ribosyl)-ATP (PRATP) and pyrophosphate (PP_i_), catalyzed by the allosteric enzyme ATP phosphoribosyltransferase (ATPPRT)^9^ (EC 2.4.2.17) (Scheme 1). The overall reaction equilibrium strongly favors the reactants.^10^ Histidine allosterically inhibits ATPPRT in a negative feedback control mechanism.^11^ In long-form ATPPRTs, both catalytic N-terminal and regulatory C-terminal domains are found in the same polypeptide chain, HisG_L_, which assembles and functions as a homohexamer in solution.^12−14^ Conversely, in short-form ATPPRTs, two distinct polypeptide chains are involved in the reaction.^15^ The catalytic subunit, HisG_S_, harbors the active site, possesses a low catalytic rate on its own, and is insensitive to histidine inhibition.^16,17^ The regulatory subunit, HisZ, a catalytically inactive paralogue of histidyl-tRNA synthetase,^15^ allosterically enhances catalysis by HisG_S_ in the absence of histidine, but in the presence of the final product of the pathway, it can bind histidine to mediate allosteric inhibition of the reaction.^15−18^ HisZ and HisG_S_ assemble in a hetero-octameric holoenzyme^19^ where a tetrameric HisZ core is sandwiched by two dimers of HisG_S_.^17,20,21^

Following the short-form ATPPRT architecture, *A. baumannii* nonactivated HisG_S_ (*Ab*HisG_S_) binds HisZ (*Ab*HisZ) to form the activated hetero-octameric holoenzyme (*Ab*ATPPRT), which leads to a substantial enhancement of the steady-state catalytic constant (*k*_cat_).^22^ Unique among ATPPRTs hitherto studied, where either ATP^23,24^ or PRPP^25,26^ must be the first substrate to bind to the free enzyme in an ordered mechanism, *Ab*ATPPRT follows a rapid equilibrium random kinetic mechanism of substrate binding, whereas product dissociation is similar to other orthologues in which PRATP is the last product to dissociate.^22^ A cold-adapted bacterium also from the Moraxellaceae family, *Psychrobacter arcticus*, possesses an orthologous short-form ATPPRT whose HisG_S_ and HisZ subunits share 69% and 43% amino acid sequence identity, respectively, with *Ab*HisG_S_ and *Ab*HisZ; despite these similarities, *P. arcticus* ATPPRT follows a strictly ordered mechanism with PRPP binding to the free enzyme.^18,25^

An in-depth kinetic investigation of allosteric regulation of catalysis is necessary to uncover fundamental aspects of this widespread phenomenon in protein biochemistry^27,28^ and to offer additional opportunities for drug design.^22,29^ The common classification of allosteric control into *K*-type (where the Michaelis constant is affected) and *V*-type (where *k*_cat_ is affected) systems,^27^ while useful at a macroscopic level, does not provide insight into the microscopic steps along the enzymatic reaction being directly perturbed by the allosteric effector.^26,30^ For example, in *Mycobacterium tuberculosis* α-isopropylmalate synthase, the first enzyme of leucine biosynthesis,^31^ where product release is the rate-limiting step in the absence of leucine, allosteric inhibition by leucine slows down the chemical step, causing it to become rate-limiting.^30^ In nonactivated *P. arcticus* HisG_S_, chemistry is rate-limiting for *k*_cat_, while in allosterically activated *P. arcticus* ATPPRT, chemistry is fast and PRATP release becomes rate-limiting.^26^ Interestingly, allosteric inhibition of *P. arcticus* ATPPRT by histidine again causes chemistry to become rate-limiting.^18^

To provide a three-dimensional structural view of *Ab*ATPPRT and to reveal in which reaction steps allosteric activation manifests itself, we employed protein crystallography, pH-rate profile, solvent viscosity effects, alternative substrate kinetics, and pre-steady-state kinetics under multiple- and single-turnover conditions. The results unveil the intricacies of catalysis and allostery in *Ab*ATPPRT and may help inform inhibitor design.

## Materials and Methods

### Materials

All chemicals were used without further purification or modification. MgCl_2_, MnCl_2_, dithiothreitol (DTT), tricine, glycerol, lysozyme, ampicillin, kanamycin, ATP, ADP, PRPP, 2-hydroxy-3-morpholinopropanesulfonic acid (MOPSO), *N*-(1,1-dimethyl-2-hydroxyethyl)-3-amino-2-hydroxypropanesulfonic acid (AMPSO), 4-(2-hydroxyethyl)piperazine-1-ethanesulfonic acid (HEPES), piperazine-*N,N*′-bis(2-ethanesulfonic acid) (PIPES), *N*-cyclohexyl-2-aminoethanesulfonic acid (CHES), polyethylene glycol 8000 (PEG-8000), and imidazole were purchased from Merck. Ethylenediaminetetraacetic acid-free complete protease inhibitor was purchased from Roche. Isopropyl β-d-1-thiogalactopyranoside and NaCl were purchased from Formedium. All other chemicals were purchased from readily available commercial sources. *Ab*HisG_S_, *Ab*HisZ, and *Mycobacterium tuberculosis* pyrophosphatase (*Mt*PPase) were obtained as previously described.^17,22^ PRATP was produced as previously reported.^32^

### Protein Crystallography

*Ab*HisG_S_ and *Ab*HisZ were mixed in a 1:1 molar ratio and buffer exchanged into 20 mM Tris pH 7.0, 50 mM KCl, and 10 mM MgCl_2_, and *Ab*ATPPRT was then concentrated to 8 mg mL^–1^ (138 μM). Crystals of *Ab*ATPPRT were grown at room temperature by hanging drop vapor diffusion by mixing protein and precipitant (0.2 M sodium nitrate, 0.1 M bis-tris propane pH 8.5, and 20% polyethylene glycol 3350) in a 1:1 molar ratio. Crystals were cryoprotected in mother liquor containing 20% glycerol (v/v) prior to flash freezing in liquid nitrogen. X-ray diffraction data were collected in house using a Rigaku 007HFM rotating anode X-ray generator coupled to a Rigaku Saturn 944+ CCD detector. Data were processed with iMosflm^33^ and scaled with Aimless.^34^ The *Ab*ATPPRT structure was solved by molecular replacement in Phaser^35^ using individual subunit structures from *Pa*ATPPRT apoenzyme (PDB ID 5M8H)^17^ as search models. The structure was refined using cycles of model building with COOT^36^ and refinement with Refmac.^37^ Some amino acid side chains and short loop sections were omitted from the model due to poor electron density. Some additional electron density was observed near the interface between *Ab*HisZ and *Ab*HisG_S_ subunits, but it could not unambiguously be identified.

### Activity Assays at 25 and 5 °C

Unless stated otherwise, initial rates at 25 °C were performed in the forward direction in 100 mM tricine pH 8.5, 15 mM MgCl_2_, 100 mM KCl, 4 mM DTT, and 10 μM *Mt*PPase. Either PRATP or *N*^1^-(5-phospho-β-d-ribosyl)-ADP (PRADP) formation was monitored by the increase in absorbance at 290 nm (ε_290_ = 3600 M^–1^ cm^–1^)^38^ over 60 s with readings every 1 s in 1 cm path-length quartz cuvettes (Hellma) in a Shimadzu UV-2600 spectrophotometer outfitted with a CPS unit for temperature control. Reactions (500 μL) were incubated for 3 min at 25 °C before being initiated by the addition of PRPP. Two independent measurements were carried out. Unless stated otherwise, initial rates at 5 °C were obtained by monitoring the increase in absorbance at 290 nm due to PRATP formation in an Applied Photophysics SX-20 stopped-flow spectrophotometer outfitted with a 5 μL mixing cell (0.5 cm path length and 0.9 ms dead time) and a circulating water bath for temperature control. One syringe contained all proteins (*Ab*HisG_S_, *Mt*PPase, and *Ab*HisZ where applicable) and ATP, while the other contained PRPP. Both syringes contained 100 mM tricine pH 8.5, 100 mM KCl, 15 mM MgCl_2_, and 4 mM DTT. Reactions were triggered by rapidly mixing 55 μL from each syringe and monitored for 60 s. A minimum of three traces with 120 data points each were collected. In all steady-state kinetics experiments described below, control reactions in the absence of *Ab*HisG_S_, *Ab*HisZ, ATP, and PRPP were carried out. Furthermore, controls were conducted to ensure that rates were independent of *Mt*PPase concentration. This was ascertained empirically by increasing the *Mt*PPase concentration in the assay, confirming that the rates did not change, and by confirming that rates are dependent on *Ab*HisG_S_ and *Ab*ATPPRT concentration. In all kinetic assays involving *Ab*ATPPRT, the *Ab*HisZ concentration was saturated based on the *K*_D_, such that the *Ab*HisZ-bound concentration of *Ab*HisG_S_ is indistinguishable from the total *Ab*HisG_S_ concentration used.

### Apparent Equilibrium Dissociation Constant (*K*_D_) for *Ab*ATPPRT at 5 °C

The *K*_D_ for *Ab*ATPPRT was determined by measuring initial rates of *Ab*HisG_S_ (0.04 μM) in the presence of 1.4 mM ATP, 1.0 mM PRPP, and varying concentrations of *Ab*HisZ (0–0.5 μM). Two independent measurements were performed.

### Dependence of *Ab*ATPPRT *k*_cat_ on pH

The concentration of a stock solution of PRATP was determined in 10 mM HEPES pH 7.5 (ε_290_ = 2800 M^–1^ cm^–1^).^38^ This stock solution was in turn diluted into either 200 mM HEPES pH 7.0 or 200 mM AMPSO pH 9.0. The pH of the final PRATP solutions was measured to ensure that they remained at the desired pH. The ε_290_ for PRATP at pHs 7.0 and 9.0 was determined by measuring the absorbance (NanoDrop) at 290 nm of known concentrations of PRATP (0.453, 0.906, and 1.812 mM at pH 7.0; 0.741, 1.234, and 2.057 mM at pH 9.0). Controls were prepared by the same procedure but in the absence of PRATP, and their absorbance at 290 nm was subtracted from the corresponding value with PRATP. Three independent measurements were carried out.

The pH dependence of *k*_cat_ was assessed by measuring initial rates of PRATP formation at 25 °C in a composite buffer system of 100 mM PIPES, 100 mM tricine, 100 mM CHES, 15 mM MgCl_2_, 100 mM KCl, and 4 mM DTT, pH 7.0–9.5 (with increments of 0.5 pH units) in the presence of 14 μM *Mt*PPase, either 0.08 μM (pH 7.0–7.5) or 0.04 μM (pH 8.0–9.5) *Ab*HisG_S_, 2 μM *Ab*HisZ, and either 1.6 mM ATP and varying concentrations of PRPP (0.4–2.5 mM) or 1.6 mM PRPP and varying concentrations of ATP (0.4–2.5 mM). The pH dependence of *Ab*ATPPRT *K*_D_ was determined from initial rates of PRATP formation in the same buffer system and pH range in the presence of 1.6 mM ATP, 1.0 mM PRPP, either 0.08 μM (pH 7.0–7.5) or 0.04 μM (pH 8.0–9.5), and varying *Ab*HisZ concentrations (0–2 μM). The following published ε_290_ were used: 2800 M^–1^ cm^–1^ (pH 7.5), 3200 M^–1^ cm^–1^ (pH 8.0), 3600 M^–1^ cm^–1^ (pH 8.5), and 4000 M^–1^ cm^–1^ (pH 9.5).^38^ To confirm enzyme stability at the extremes of the pH range, *Ab*HisG_S_ and *Ab*HisZ were incubated independently at pH 7.0 and 9.5 for 25 min at 4 °C prior to activity measurement at pH 8.5 in the presence of 1.6 mM ATP and 1.6 mM PRPP, without any change in activity. The concentration of *Mt*PPase was doubled at the extremes of the pH range without any effect on the measured *Ab*ATPPRT initial rate with 1.6 mM PRPP and 1.6 mM ATP. Two independent measurements were carried out.

### *Ab*HisG_S_ and *Ab*ATPPRT Substrate Saturation Kinetics

Substrate saturation curves for PRPP and ATP were determined at 5 and 25 °C by measuring initial rates with either 3 μM and 1 μM *Ab*HisG_S_ (at 5 and 25 °C, respectively) or 0.039 μM *Ab*ATPPRT (0.04 μM *Ab*HisG_S_ and 2 μM *Ab*HisZ) at a fixed concentration of one substrate (either 1.6 mM and 3.2 mM PRPP for *Ab*ATPPRT and *Ab*HisG_S_, respectively, or 1.6 mM and 6.4 mM ATP for *Ab*ATPPRT and *Ab*HisG_S_, respectively) and varying concentrations of the cosubstrate (either 0–1.6 mM and 0–3.2 mM PRPP for *Ab*ATPPRT and *Ab*HisG_S_, respectively, or 0–1.6 mM and 0–6.4 mM for *Ab*ATPPRT and *Ab*HisG_S_, respectively). Substrate saturation curves for ADP were determined at 25 °C under identical conditions, except that ADP was used instead of ATP. Two independent measurements were carried out.

### *Ab*HisG_S_ Substrate Saturation Kinetics with MnCl_2_

Owing to the presence of magnesium in the *Ab*HisG_S_ storage buffer,^22^*Ab*HisG_S_ was dialyzed against 2 × 1 L of 10 mM Tris-HCl and 100 mM NaCl pH 8.0 before enzymatic assays. Substrate saturation curves were determined as described above but in the presence of 15 mM MnCl_2_ instead of MgCl_2_. Data for *Ab*HisG_S_ were collected with ADP and ATP at 25 °C but with only ATP at 5 °C. Two independent measurements were carried out. Several attempts to determine the kinetics of *Ab*ATPPRT at 5 and 25 °C in the presence of 15 mM MnCl_2_ instead of MgCl_2_ were unsuccessful, with MnCl_2_ leading to severe inhibition of the reaction.

### *Ab*HisG_S_ and *Ab*ATPPRT Pre-Steady-State Kinetics

The approach to the steady state for the *Ab*HisG_S_ and *Ab*ATPPRT reactions was investigated under multiple- and single-turnover conditions at 290 nm in an Applied Photophysics SX-20 stopped-flow spectrophotometer outfitted with a 5 μL mixing cell (0.5 cm path length and 0.9 ms dead time) and a circulating water bath for temperature control. Each syringe contained 100 mM tricine pH 8.5, 100 mM KCl, 15 mM MgCl_2_, and 4 mM DTT. Reactions were triggered by rapidly mixing 55 μL from each syringe. For each reaction, including controls, a minimum of five traces were collected under multiple-turnover conditions, and six traces under single-turnover conditions.

For multiple-turnover experiments at 25 °C, one syringe carried 20 μM *Ab*HisG_S_, 60 μM *Mt*PPase, and 12.6 mM ATP, and the other contained 12 mM PRPP. Alternatively, one syringe carried 20 μM *Ab*HisG_S_, 30 μM *Ab*HisZ, 60 μM *Mt*PPase, and 3.2 mM ATP, and the other contained 3.2 mM PRPP. PRATP formation was monitored for 5 s with 4000 data points collected in the *Ab*HisG_S_ reaction and for 0.5 s with 4000 data points collected for the *Ab*ATPPRT reaction. For *Ab*HisG_S_ reactions at 5 °C, one syringe carried 20 μM *Ab*HisG_S_, 25 μM *Mt*PPase, and 12.6 mM ATP, and the other contained 6.4 mM PRPP. PRATP formation was monitored for 8 s with 4000 data points collected. For *Ab*ATPPRT reactions at 5 °C, one syringe contained 20 μM *Ab*ATPPRT, 60 μM *Mt*PPase, and 3.2 mM ATP, and the other contained 3.2 mM PRPP. PRATP formation was monitored for 1 s in a split-time base with 4000 data points collected for the first 0.5 s and 2000 data points for the remaining time. Controls lacked PRPP.

For *Ab*ATPPRT single-turnover kinetics at 5 °C, *Ab*HisG_S_ and *Ab*HisZ were initially mixed in a 0.8:1 molar ratio and buffer-exchanged into 100 mM tricine pH 8.5, 100 mM KCl, 15 mM MgCl_2_, and 4 mM DTT, concentrated using a Vivaspin (Millipore), and the *Ab*ATPPRT concentration determined at 280 nm (NanoDrop) using the molar-ratio-weighted sum of the ε_280_ for *Ab*HisG_S_ and *Ab*HisZ^22^ (51 664 M^–1^ cm^–1^). One syringe contained 12 μM PRPP, 40 μM *Mt*PPase, and either 150 μM or 200 μM *Ab*ATPPRT, and the other syringe contained 3.2 mM ATP. PRATP formation was monitored for 0.11 s with 8000 data points collected per trace. Controls lacked PRPP and were subtracted from the reactions containing PRPP. A significant lag time was observed in all traces, and the data could only be fitted after the first 0.01 s were removed from all traces.

### *Ab*ATPPRT Kinetics in the Presence of Glycerol

*Ab*ATPPRT initial rates at 25 °C were measured at a saturating concentration of ATP (1.6 mM) and two saturating concentrations of PRPP (1.4 and 1.6 mM) in the presence of 0–12% glycerol (v/v). Controls in 12% glycerol were performed in the presence of both 10 μM and 15 μM *Mt*PPase to ensure that the rate was not dependent on *Mt*PPase. The *Ab*ATPPRT *K*_D_ in 12% glycerol was determined by measuring initial rates of 0.08 *Ab*HisG_S_ in the presence of 1.6 mM ATP, 1.6 mM PRPP, and varying concentrations of 0–0.5 μM *Ab*HisZ. *Ab*ATPPRT initial rates at 25 °C were also measured at a saturating concentration of ATP (1.6 mM) and two saturating concentrations of PRPP (1.4 and 1.6 mM) in the presence of 5% PEG-8000 (v/v). Two independent measurements were performed.

### Kinetics Data Analysis

Kinetics data were analyzed by the nonlinear regression function of SigmaPlot 13.0 (SPSS Inc.). Data points and error bars represent mean ± SEM, and kinetic and equilibrium constants are given as mean ± fitting error. Initial rate data at varying concentrations of *Ab*HisZ were fitted to eq 1, and the concentration of *Ab*ATPPRT at any concentration of *Ab*HisG_S_ and *Ab*HisZ was calculated with eq 2. Substrate saturation curves at a fixed concentration of the cosubstrate were fitted to eq 3. The pH-rate profile was fitted to eq 4. Solvent viscosity effects were fitted to eq 5. Pre-steady-state kinetics data under multiple-turnover conditions were fitted either to eq 6 or to eq 7, and under single-turnover conditions, to eq 8. In eqs 1–8, *v* is the initial rate, *k*_cat_ is the apparent steady-state catalytic rate constant, *K*_M_ is the apparent Michaelis constant, *E*_T_ is total enzyme concentration, *S* is the concentration of the varying substrate when the cosubstrate is held constant, *C* is the pH-independent value of *k*_cat_, *H* is the proton concentration, *K*_a_ is the apparent acid dissociation constant, *k*_cat_^0^ and *k*_cat_^η^ represent the *k*_cat_ in the absence and presence of glycerol, respectively, η_rel_ is the relative viscosity of the solution, *m* is the slope, *V*_max_ is the maximal velocity, *G* is the concentration of *Ab*HisG_S_, *Z* is the concentration of *Ab*HisZ, *K*_D_ is the apparent equilibrium dissociation constant, AbATPPRT is the concentration of *Ab*ATPPRT complex, *P*(*t*) is product concentration as a function of time *t*, *k* is the observed rate constant for the exponential phase, ES is the enzyme–substrate complex concentration, and *k*_2_ and *k*_3_ are rate constants governing sequential steps in a single turnover.
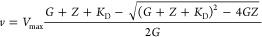
1
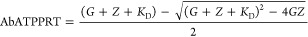
2
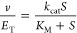
3
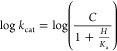
4
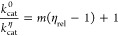
5
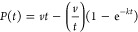
6
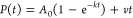
7
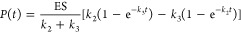
8

## Results

### *Ab*ATPPRT Crystal Structure

**Figure 1:**
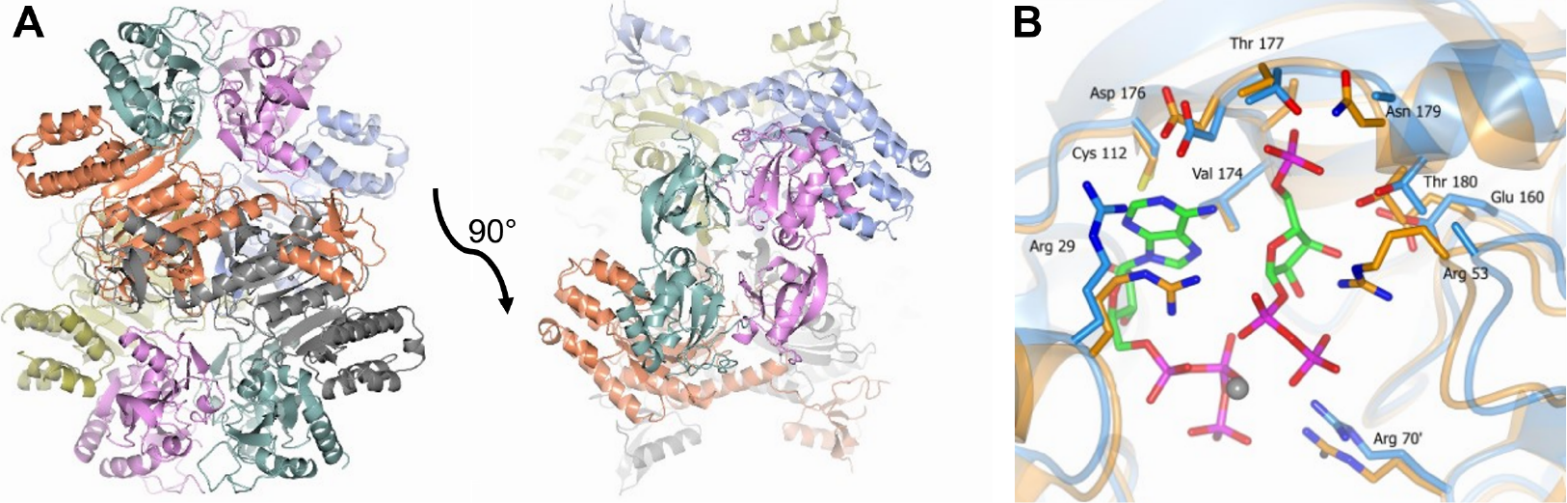
Crystal structure of the unliganded *Ab*ATPPRT. (A) Two views of the ribbon diagram of the hetero-octamer. *Ab*HisG_S_ subunits are shown in pink and teal, whereas *Ab*HisZ subunits are in gray, orange, yellow, and blue. (B) Active-site close-up of the overlay between *Ab*HisG_S_ and PRPP-ATP-bound *P. arcticus* HisG_S_ (6FU2) dimers. Side chains are shown as stick models with carbon atoms in blue for *Ab*ATPPRT and in gold for *P. arcticus* ATPPRT. Residue labels follow the *Ab*ATPPRT numbering. No electron density was visible for most of the side-chain atoms of Arg53 and Asn179. PRPP and ATP (from the *P. arcticus* structure) are shown as stick models with carbon atoms in green. The Mg^2+^ (from the *P. arcticus* structure) is shown as a sphere.

To start shedding light on the structural underpinnings of *Ab*ATPPRT catalysis and help inform structure-based inhibitor design, the three-dimensional structure of unliganded *Ab*ATPPRT was determined at 2.40 Å resolution in space group *P*2_1_, with the full hetero-octamer in the asymmetric unit. The coordinates were deposited to the Protein Data Bank (PDB ID: 8OY0). Complete data collection and refinement statistics are summarized in Table S1. The *Ab*ATPPRT hetero-octamer consists of the characteristic arrangement^17,20,21^ of four *Ab*HisZ subunits flanked on each side by an *Ab*HisG_S_ homodimer. Each subunit of the *Ab*HisG_S_ homodimer interacts in a head-to-tail arrangement with the two active sites located at each site of a crevice between them. Each pair of *Ab*HisZ subunits also forms a head-to-tail homodimer (Figure 1A). The overall structure resembles that of *P. arcticus* ATPPRT (PDB ID: 5M8H),^17^ and overlay of the two hetero-octamers (Figure S1A) yielded a root-mean-square deviation (RMSD) of 3.50 Å over 2113 Cα atoms.

Overlay of the *Ab*ATPPRT structure with *Lactococcus lactis* ATPPRT bound to PRPP (PDB ID: 1Z7M)^21^ (Figure S1B) and with *Thermotoga maritima* ATPPRT bound to histidine (PDB ID: 1USY)^20^ (Figure S1C) yielded much larger RMSDs of 31.64 Å over 1973 Cα atoms and 46.56 Å over 1833 Cα atoms, respectively. These sizable RMSDs might be attributed to the presence of ligands in the structures and/or the lower sequence identity relative to *P. arcticus* ATPPRT, shared by these proteins with *Ab*ATPPRT. *Ab*HisG_S_ shares 40% and 33% amino acid sequence identity with *L. lactis* and *T. maritima* HisG_S_, respectively, and *Ab*HisZ shares 23% and 22% amino acid sequence identity with *L. lactis* and *T. maritima* HisZ, respectively. Furthermore, *L. lactis* and *T. maritima* HisZ lack the C-terminal domain present in both *Ab*HisZ and *P. arcticus* HisZ.^18^ They also reflect differences in domain conformations. When *Ab*HisG_S_ is overlaid with *T. maritima* and *L. lactis* HisG_S_, the RMSDs between *Ab*HisG_S_ and *T. maritima* HisG_S_ and *Ab*HisG_S_ and *L. lactis* HisG_S_ are only 3.38 Å over 193 Cα atoms and 2.46 Å over 192 Cα atoms, respectively; when the HisZ subunits are overlaid, the RMSDs between *Ab*HisZ and *T. maritima* HisZ and *Ab*HisG_S_ and *L. lactis* HisZ are 7.07 Å over 264 Cα atoms and 8.94 Å over 192 Cα atoms, respectively.

The *Ab*ATPPRT and PRPP-ATP-bound *P. arcticus* ATPPRT (6FU2)^25^ have an RMSD of 1.08 Å over 372 Cα atoms when the corresponding HisG_S_ dimers are overlaid. Figure 1B shows the strict amino acid sequence conservation between the two active sites, with conserved residues known to be involved in substrate binding and/or catalysis in *P. arcticus* ATPPRT^25,28^ shown for both structures. The only noticeable difference in conformation between the side chains of corresponding active-site residues is the orientation of Arg29 (Arg32 in *P. arcticus*), found in the open conformation in *Ab*ATPPRT as opposed to the closed conformation in *P. arcticus* ATPPRT, which is characteristic of the Michaelis complex.^25,28^ The open conformation is seen, however, in the unliganded form of *P. arcticus* ATPPRT.^17^ Given their remarkably similar active-site structures, it is puzzling that *P. arcticus* ATPPRT follows an ordered kinetic mechanism,^26^ while *Ab*ATPPRT follows a random one.^22^

### *Ab*ATPPRT *k*_cat_ pH-Rate Profile

The reaction catalyzed by *Ab*ATPPRT must involve the loss of a proton from the 6-NH_2_^+^ group, probably after the transition state for nucleophilic substitution on PRPP C1, to yield the 6-NH group of PRATP.^25,26^ To interrogate the role of acid–base catalysis in the reaction, the pH-rate profile of *Ab*ATPPRT was obtained. As the PRATP ε_290_ is pH-dependent and only reported for pHs 7.5–8.5 and 9.5,^38^ the values at pHs 7.0 and 9.0 were first determined to be 2550 M^–1^ cm^–1^ and 3800 M^–1^ cm^–1^, respectively (Figure S2). The pH dependence of the allosteric activation of *Ab*HisG_S_ by *Ab*HisZ was assessed (Figure S3), and best fit of the data to eq 1 yielded *K*_D_ values for the *Ab*HisG_S_–*Ab*HisZ interaction shown in Table S2. No pH dependence of the *K*_D_ was noticeable, and *Ab*ATPPRT concentration was calculated using eq 2. At pH 7.0, rates were too low to measure accurately at low *Ab*HisZ levels, so only an upper limit of less than 0.5 μM for the *K*_D_ could be estimated. Nonetheless, as the rate had essentially plateaued at 2 μM *Ab*HisZ, the *Ab*ATPPRT concentration was assumed to be that of *Ab*HisG_S_.

**Figure 2:**
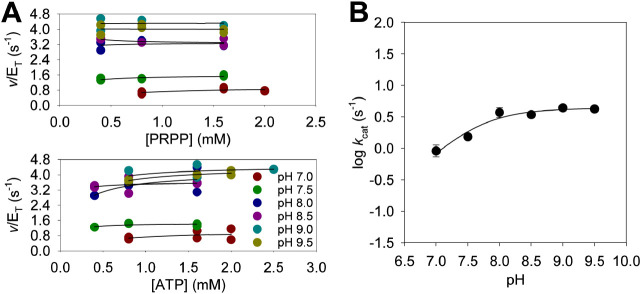
*Ab*ATPPRT pH-rate study. (A) Substrate concentration dependence of *Ab*ATPPRT initial rate at different pHs. All data points are shown for two independent measurements at each concentration. Lines are best fit to eq 3. (B) *Ab*ATPPRT pH-rate profile of *k*_cat_. Data are mean ± fitting error from two independent measurements. Line is best fit to eq 4.

Owing to the decrease in ε_290_ at the lower pHs, rates could only be accurately measured at substrate concentrations near saturation; thus, best fit to eq 3 yielded solely *k*_cat_ at different pHs (Figure 2A). The *Ab*ATPPRT pH-rate profile was best fit to eq 4 (Figure 2B), which would normally indicate that deprotonation of a group with a p*K*_a_ of 7.6 ± 0.5 is required for catalysis, possibly to act as a general base and accept a proton from the 6-NH_2_^+^ group. Nonetheless, eq 4 presumes a slope of 1 on the acidic limb,^39^ but the slope of log *k*_cat_ from pH 7.0–8.0 was only 0.65, precluding a direct mechanistic interpretation in terms of protonation state and p*K*_a_ of any specific group. It can be stated that *Ab*ATPPRT *k*_cat_ is pH independent above pH 8.0 and decreases below this pH.

### *Ab*HisG_S_ and *Ab*ATPPRT Pre-Steady-State Kinetics at 25 °C

**Figure 3:**
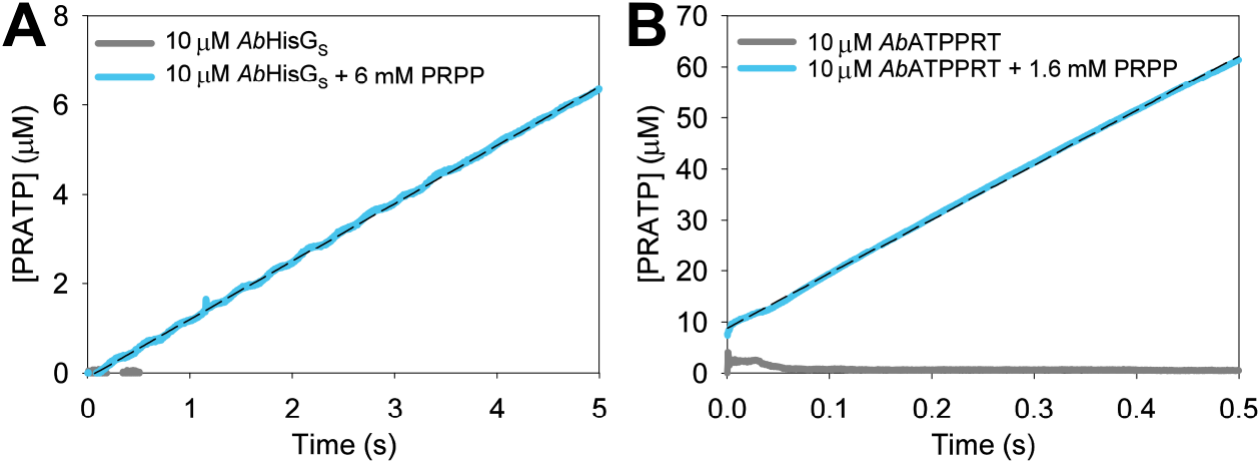
Pre-steady-state kinetics at 25 °C. (A) Approach to the steady-state formation of PRATP by *Ab*HisG_S_. (B) Approach to the steady-state formation of PRATP by *Ab*ATPPRT. Dashed lines are linear regressions of the data. Controls lacked PRPP.

To start to interrogate which steps are rate-limiting for the *Ab*HisG_S_ and *Ab*ATPPRT reactions and which steps are directly affected by allosteric activation, the approach to steady-state formation of PRATP was analyzed using rapid kinetics. The *Ab*HisG_S_ reaction showed no burst in PRATP formation (Figure 3A), and the apparent steady-state rate constant of 0.13 ± 0.01 s^–1^ is within three-fold of *k*_cat_ (Table 1). Only a linear increase in PRATP formation could be directly observed in the *Ab*ATPPRT reaction (Figure 3B), with an apparent steady-state rate constant of 10.66 ± 0.06 s^–1^, in agreement with *k*_cat_ (Table 1). However, a *y*-axis offset corresponding to ∼8.9 μM PRATP (Figure 3B) implies a burst of PRATP formation taking place within 0.9 ms (the dead time of the stopped-flow spectrophotometer). These observations suggest that on-enzyme formation of PRATP is rate-limiting in the reaction catalyzed by *Ab*HisG_S_, but a step after chemistry limits the reaction catalyzed by *Ab*ATPPRT.^40^ Similar conclusions were drawn from pre-steady-state kinetic analysis of *P. arcticus* HisG_S_ and ATPPRT, except the burst phase could be directly observed with the activated enzyme.^26^ A burst of PRATP formation was also reported for the *Mycobacterium tuberculosis* HisG_L_ reaction.^41^

**Table 1:** *Ab*HisG_S_ and *Ab*ATPPRT Steady-State Kinetics at 25 °C

enzyme	nucleotide/metal	*k*_cat_ (s^–1^)	*K*_M_^AXP^^a^ (mM)	*K*_M_^PRPP^ (mM)	*k*_cat_/*K*_M_^AXP^^a^(M^–1^ s^–1^)	*k*_cat_/*K*_M_^PRPP^ (M^–1^ s^–1^)
*Ab***HisG**_**S**_	ATP/Mg^2+^	0.384 ± 0.006	0.83 ± 0.06	0.60 ± 0.06	460 ± 30	640 ± 60
	ADP/Mg^2+^	0.48 ± 0.02	1.5 ± 0.3	1.2 ± 0.1	320 ± 70	400 ± 40
	ATP/Mn^2+^	0.94 ± 0.03	0.39 ± 0.07	0.60 ± 0.08	2400 ± 400	1600 ± 200
	ADP/Mn^2+^	3.3 ± 0.1	2.2 ± 0.3	0.44 ± 0.06	1500 ± 200	8000 ± 1000
*Ab***ATPPRT**	ATP/Mg^2+^	10.8 ± 0.3	0.19 ± 0.02	0.14 ± 0.01	57 000 ± 6000	77 000 ± 6000
	ADP/Mg^2+^	16.6 ± 0.3	0.36 ± 0.03	0.096 ± 0.007	46 000 ± 4000	170 000 ± 10 000

a X denotes either T or D.

### PRATP Diffusional Release from *Ab*ATPPRT is Rate Determining at 25 °C

**Figure 4:**
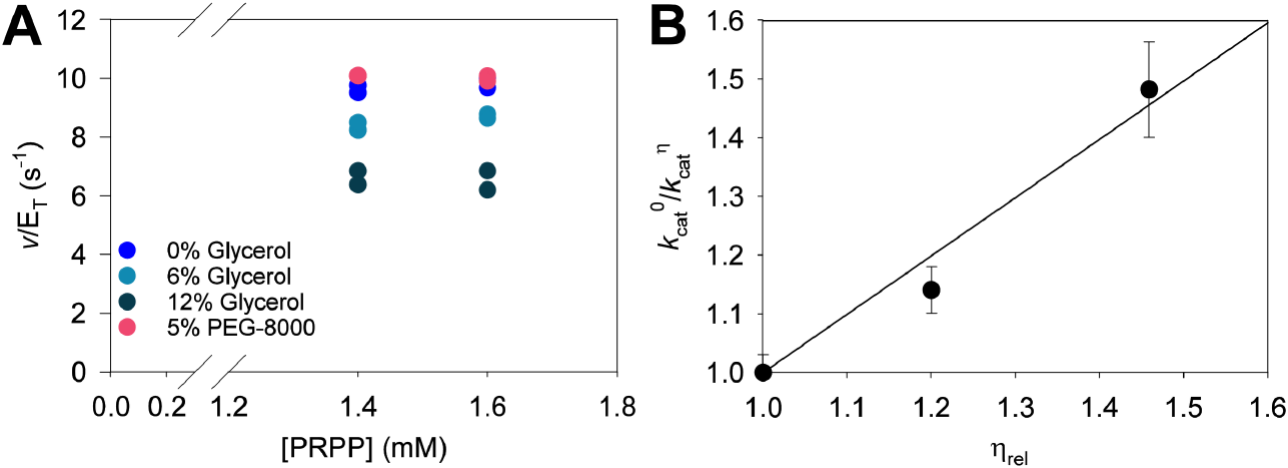
Solvent viscosity effects on the *Ab*ATPPRT-catalyzed reaction. (A) *Ab*ATPPRT apparent rate constants for PRATP formation at saturating substrate concentrations in the presence and absence of either glycerol or PEG-8000. All data points are shown for two independent measurements at each concentration. (B) Solvent viscosity effects on *k*_cat_. Data are mean ± SD for four independent measurements (two at each concentration). Line is best fit to eq 5.

The inferred burst of PRATP formation with *Ab*ATPPRT may indicate that product release limits *k*_cat_. To test this hypothesis and assess whether product release itself is limited by product diffusion from the enzyme–product complex, *Ab*ATPPRT rates at saturating concentrations of substrates were measured in the presence and absence of glycerol (Figure 4A). *Ab*ATPPRT rates were insensitive to the macroviscogen PEG-8000 (Figure 4A), suggesting that any effect with glycerol is the result of increased solvent microviscosity.^42^ Furthermore, increasing *Mt*PPase concentration had no effect on *Ab*ATPPRT rates in 12% glycerol (Figure S4A), and determination of the *Ab*ATPPRT *K*_D_ in 12% glycerol (Figure S4B) yielded a value in agreement to that previously published.^22^ A plot of *k*_cat_ ratios versus relative viscosity (Figure 4B) resulted in a slope of 0.99 ± 0.09. This is within the experimental error of the maximum theoretical value for this type of plot and indicates that product diffusion from *Ab*ATPPRT is rate determining for *k*_cat_.^42^ Given the very low affinity of a PP_i_ analogue for the *Ab*ATPPRT:PRATP complex (*K*_D_ of ∼8 mM) and the comparably high affinity of PRATP for *Ab*ATPPRT (*K*_D_ of ∼25 μM),^22^ it is probable that PP_i_ release from the *Ab*ATPPRT:PRATP:PP_i_ ternary complex is fast, and diffusion of PRATP from the *Ab*ATPPRT:PRATP binary complex determines the overall catalytic rate. Solvent viscosity effects also indicated that product diffusion limits *P. arcticusAb*ATPPRT *k*_cat_.^26^

### ADP is a Substrate of *Ab*HisG_S_ and *Ab*ATPPRT

**Figure 5:**
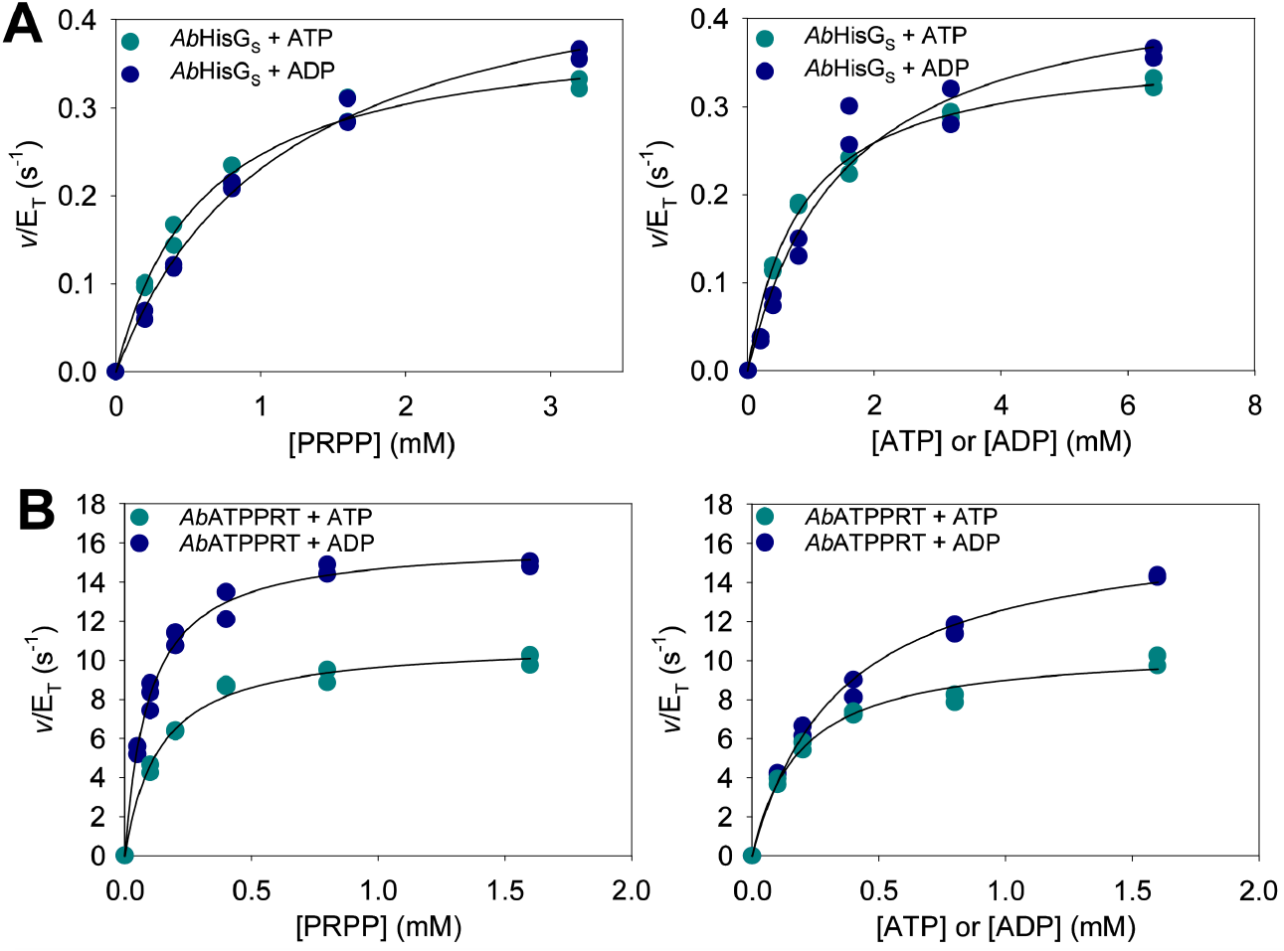
Steady-state kinetics with ADP. (A) Substrate concentration-dependence of *Ab*HisG_S_ initial rates with either ATP or ADP as substrate. (B) Substrate concentration-dependence of *Ab*ATPPRT initial rates with either ATP or ADP as substrate. All data points are shown for two independent measurements at each concentration. Lines are best fit to eq 3.

For long-form ATPPRTs, ADP acts as a competitive inhibitor against ATP.^43^ Conversely, for *P. arcticus* HisG_S_, ADP is an alternative substrate to ATP with comparable kinetics.^26^ ATP and ADP bind in a remarkably similar way to *P. arcticus* ATPPRT, except for the γ-PO_4_^2–^ group of ATP (and PRATP) which forms a salt-bridge with the conserved Arg73 side chain (Arg70 in *Ab*ATPPRT), an interaction which is absent in the case of ADP.^25^ If a salt-bridge between ATP (or PRATP) γ-PO_4_^2–^ and *Ab*ATPPRT Arg70 is operational, its absence when ADP is the substrate might facilitate release of *N*^1^-(5-phospho-β-d-ribosyl)-ADP ever so slightly, which would be reflected in a modest but significant increase in *Ab*ATPPRT *k*_cat_, since product release is rate determining. On the contrary, *Ab*HisG_S_*k*_cat_ would not be expected to change significantly if chemistry were rate-limiting in this case as the γ-PO_4_^2–^ group is relatively far from the reacting groups. This may also be accompanied by an increase in *K*_M_ of the nucleotide, as the contact between ATP γ-PO_4_^2–^ and Arg70 would be expected to contribute to substrate binding. To test these hypotheses, ADP was evaluated as a substrate of *Ab*HisG_S_/*Ab*ATPPRT. *Ab*HisG_S_ accepts ADP as a substrate with very similar kinetics (Figure 5A) and PRPP specificity constants (*k*_cat_/*K*_M_^PRPP^) to ATP, except for an ∼two-fold increase in *K*_M_^ADP^ as compared with *K*_M_^ATP^ (Table 1). In the case of *Ab*ATPPRT, replacement of ATP for ADP (Figure 5B) increases both *k*_cat_ and *k*_cat_/*K*_M_^PRPP^, which is also accompanied by an almost two-fold increase in *K*_M_^ADP^ as compared with *K*_M_^ATP^ (Table 1). These observations lend further support to *Ab*ATPPRT *k*_cat_ reflecting the rate of PRATP departure from the active site, whereas *Ab*HisG_S_*k*_cat_ reflects on-enzyme chemistry.

### Mn^2+^ Enhances *Ab*HisG_S_ Catalysis at 25 °C

**Figure 6:**
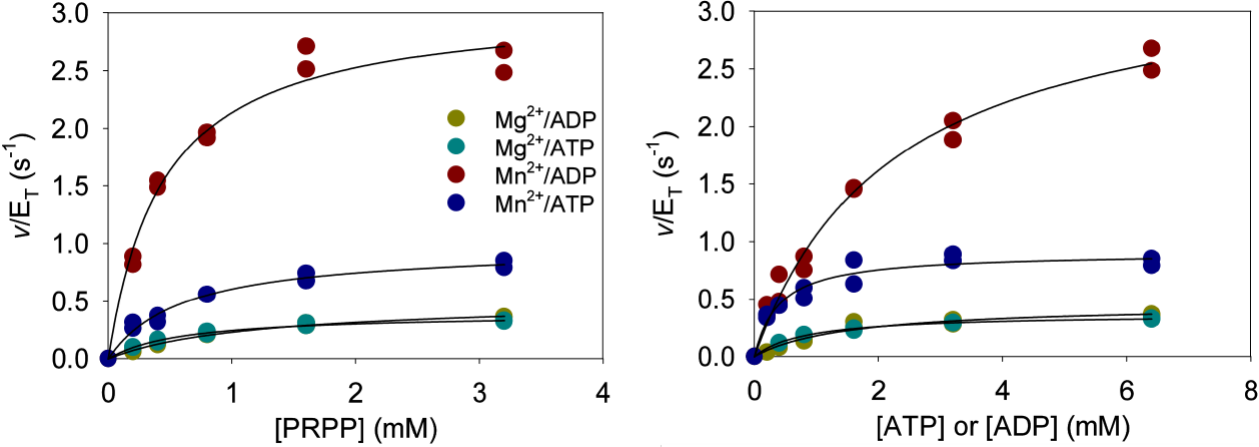
*Ab*HisG_S_ steady-state kinetics with Mn^2+^ at 25 °C. All data points are shown for two independent measurements at each concentration. Lines are best fit to eq 3.

Mg^2+^ is proposed to facilitate ATPPRT catalysis by acting as a Lewis acid to offset the negative charge build-up in the PP_i_ leaving group at the transition state.^26,44^ In *P. arcticus* HisG_S_, replacement of Mg^2+^ by Mn^2+^ increases *k*_cat_, which is interpreted as evidence that chemistry is the rate-limiting step since Mn^2+^ serves as a better Lewis acid at the transition state.^26^ In long-form ATPPRTs, where chemistry is fast and product release limits *k*_cat_,^41,44^ the effect of Mn^2+^ is either null or inhibitory.^10,45^ To evaluate if chemistry is rate limiting for *Ab*HisG_S_*k*_cat_, a hypothesis derived from the lack of burst of PRATP formation with the nonactivated enzyme, the effect of Mn^2+^ on *Ab*HisG_S_ kinetics was assessed. Use of Mn^2+^ instead of Mg^2+^ causes a 2.4-fold and a 6.7-fold increase in *Ab*HisG_S_*k*_cat_ with ATP and ADP as substrates, respectively (Figure 6 and Table 1). This observation supports the hypothesis that chemistry is the rate-limiting step in *Ab*HisG_S_ catalysis.

### Multiple-Turnover Pre-Steady-State Kinetics at 5 °C

**Figure 7:**
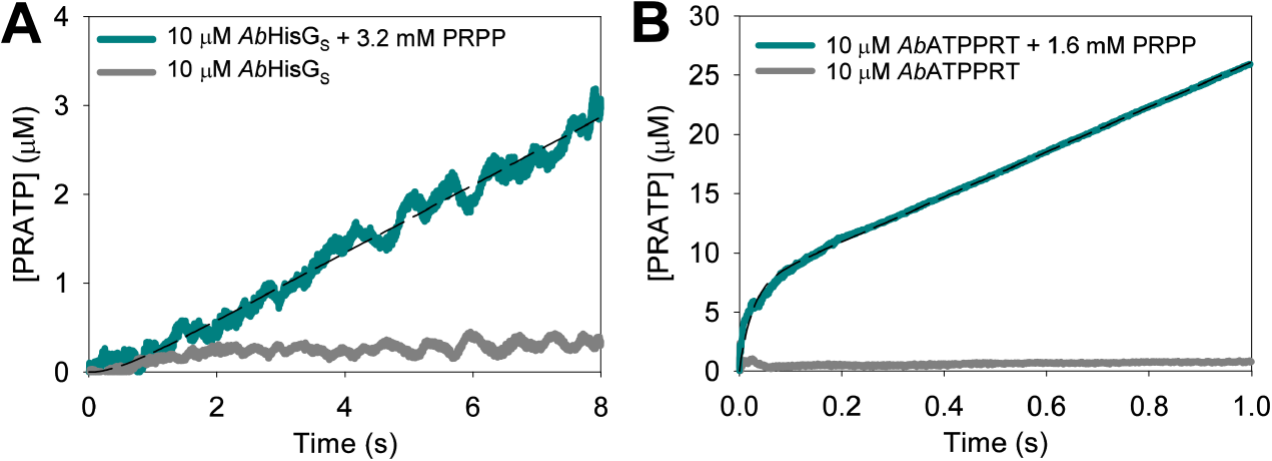
Pre-steady-state kinetics at 5 °C. (A) Approach to the steady-state formation of PRATP by *Ab*HisG_S_. The dashed line is the best fit to eq 6. (B) Approach to the steady-state formation of PRATP by *Ab*ATPPRT. The dashed line is the best fit to eq 7. Controls lacked PRPP.

To slow down the reaction and attempt to capture the first turnover of *Ab*ATPPRT on its approach to the steady state, *Ab*HisG_S_ and *Ab*ATPPRT pre-steady-state kinetics were evaluated at 5 °C (Figure 7). Again, no burst phase was present with *Ab*HisG_S_ (Figure 7A), indicating that steps after chemistry are fast,^40^ but an apparent lag time preceding the approach to the steady state was observed. Best fit of the data to eq 6 yielded an apparent steady-state rate constant of 0.038 ± 0.001 s^–1^, close to the *Ab*HisG_S_*k*_cat_ (Table S3) obtained from substrate saturation curves at 5 °C (Figure S5), and an observed rate constant for the exponential approach to the steady state of 2.09 ± 0.06 s^–1^. Such a lag phase is not uncommon in mechanisms lacking a burst of product formation,^46^ having also been reported, for example, for *Trypanosoma cruzi* uridine phosphorylase.^47^ Moreover, a 4.5-fold increase in *Ab*HisG_S_*k*_cat_ upon replacement of Mg^2+^ by Mn^2+^ (Figure S6, Table S3) suggests that chemistry is also rate limiting at 5 °C.

Allosteric activation of *Ab*HisG_S_ by *Ab*HisZ at 5 °C resulted in a *K*_D_ of 41 ± 4 nM (Figure S7A). The steady-state kinetics of *Ab*ATPPRT at 5 °C (Figure S7B) resulted in the kinetic parameters summarized in Table S3. At 5 °C, a burst of PRATP production can be seen preceding the steady-state phase of the *Ab*ATPPRT reaction (Figure 7B), suggesting a step after chemistry limits the reaction rate,^40^ and best fit of the data to eq 7 yielded an observed rate constant for the burst phase of 39.3 ± 0.3 s^–1^, a burst-phase amplitude of 7.1 μM, approaching the concentration of enzyme (10 μM), and a steady-state rate constant of 1.90 ± 0.02 s^–1^, in reasonable agreement with *Ab*ATPPRT *k*_cat_ (3.0 s^–1^) (Table S3).

### *Ab*ATPPRT Single-Turnover Kinetics at 5 °C

**Figure 8:**
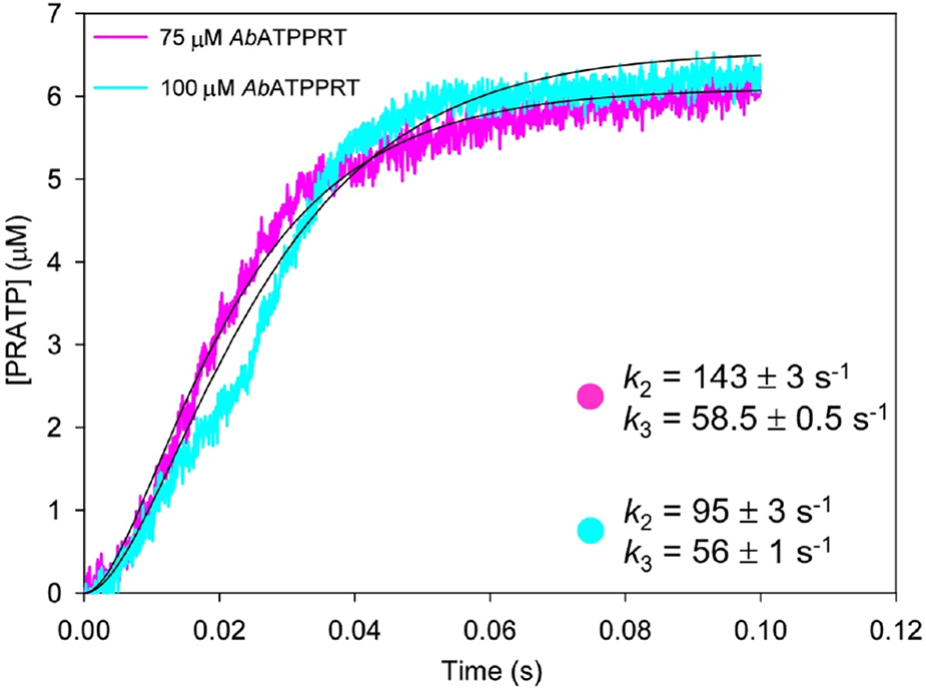
Single-turnover kinetics of *Ab*ATPPRT at 5 °C. Black lines are best fit of the data to eq 8, which yielded the apparent rate constants shown.

To gather additional information on the rate of on-enzyme PRATP synthesis, *Ab*ATPPRT catalysis was analyzed under single-turnover conditions with PRPP (6 μM) as the limiting substrate. As 20 μM *Mt*PPase are available to hydrolyze a maximum of only ∼6 μM PP_i_, the excess *Mt*PPase will render the reaction essentially irreversible if PP_i_ dissociates from the *Ab*ATPPRT:PRATP:PP_i_ complex faster than this complex is converted back to *Ab*ATPPRT:PRPP:ATP. The overall single-turnover amplitudes indicate that ∼6 μM PRATP was produced (Figure 8), consistent with the aforementioned scenario. A short lag time in PRATP formation was observed, and the data could only be satisfactorily fitted with eq 8 (Figure 8), which describes two consecutive irreversible steps to product formation. These could be a binding step followed by chemistry or an isomerization of the Michaelis complex followed by chemistry. Increasing the enzyme concentration did not lead to an increase in the rate constants, which is compatible with a unimolecular process^46^ where all the PRPP is bound to ATPPRT at the beginning of the reaction. This favors a mechanism where the Michaelis complex isomerizes before PRATP is formed. All rate constants (Figure 8) are much higher than *k*_cat_ (Table S3), suggesting that the chemical step is faster than a subsequent step, consistent with the observed burst of PRATP formation.

It should be pointed out that, in lieu of a signal for the absolute amplitude of the isomerization of the Michaelis complex, it is not possible to ascertain unambiguously which of the consecutive irreversible steps is governed by *k*_2_ and which is governed by *k*_3_. Mathematical simulations have demonstrated both orders of events, i.e., fast step followed by slow step and fast step preceding slow step, produce identical transients for product formation.^48^

## Discussion

Dissecting the kinetics of allosteric regulation of enzymes is crucial to understand which steps along the reaction cycle are responding to the allosteric effector.^26,28,30,49^ In addition, recent studies have highlighted the effect temperature can exert on allosteric regulation,^50,51^ and how dynamic allostery responds to temperature changes to drive temperature adaptation.^49^ As an example, in thermophilic *T. maritima* imidazole glycerol phosphate synthase (IGPS), a *V*-type heterodimeric allosteric enzyme catalyzing the fifth step of histidine biosynthesis, allosteric activation by *N*′-[5′-phosphoribulosyl)formimino]-5-aminoimidazole-4-carboxamide-ribonucleotide leads to 4200-fold increase in *k*_cat_ at 30 °C, but only 65-fold at 70 °C (near *T. maritima*’s natural growth temperature).^50^ This was explained in terms of temperature-activated protein motions that mimic those observed upon allosteric activation.^51^

*Ab*ATPPRT is predominantly a *V*-type enzyme where allosteric activation of *Ab*HisG_S_ by *Ab*HisZ causes ∼73- and ∼29-fold enhancement in *k*_cat_ at 5 and 25 °C, respectively, in qualitative agreement with the trend reported for *T. maritima* IGPS.^50^ However, the present work also furnished evidence that while the chemical step is rate-limiting for *Ab*HisG_S_*k*_cat_, it is disproportionately activated by allosteric binding of *Ab*HisZ, rendering product release rate-limiting for *Ab*ATPPRT *k*_cat_. To appreciate the magnitude of allosteric activation of the chemical step, one must compare *Ab*HisG_S_*k*_cat_ with the single-turnover rate constant for *Ab*ATPPRT. If the lowest of these single-turnover rate constants (the lower *k*_3_ value in Figure 8) are assumed to govern the chemical step in *Ab*ATPPRT, allosteric modulation results in ∼1356-fold speed-up of chemistry at 5 °C. At 25 °C, chemistry becomes so fast with *Ab*ATPPRT that the first on-enzyme turnover takes place within 0.9 ms. Speculatively, this would imply a single-turnover rate constant of at least ∼1111 s^–1^, which would mean an allosteric activation of the chemical step of ∼2893-fold, the opposite temperature-dependence trend of allosteric activation of *k*_cat_.

*Ab*ATPPRT is a promising target for novel antibiotic discovery against *A. baumannii*-caused pneumonia. In fact, recent work carried out *in-silico* screening of inhibitors of *Ab*HisG_S_ based on a homology model built with *P. arcticus* HisG_S_ as a template.^52^ Intriguingly, while genes encoding *Ab*HisG_S_ and other enzymes of the histidine biosynthetic pathway have been shown to be necessary for *A. baumannii* persistence in the lungs of mice,^7,8^ the gene encoding *Ab*HisZ was proposed to be essential for survival even in rich medium based on high-throughput transposon library analysis.^7^ Here, we reported the crystal structure of *Ab*ATPPRT, which will enable structure-based design of orthosteric and allosteric inhibitors of this enzyme. Furthermore, it could inform the design of chemical probes to disrupt the interaction between *Ab*HisG_S_ and *Ab*HisZ to investigate the role of *Ab*HisZ in *A. baumannii* survival. Finally, the demonstration that PRATP diffusion from *Ab*ATPPRT is the kinetic bottleneck for *k*_cat_ may help inform inhibitor discovery strategies. For example, it may be desirable to screen compounds against *Ab*ATPPRT in the presence of high concentrations of PRATP (or both PRPP and ATP), to increase the probability of finding potent hits toward the most stable form of the enzyme, the *Ab*ATPPRT:PRATP complex.

## Supporting Information Available

Further results and analysis of *Ab*ATPPRT catalysis (PDF)

## Accession Codes

*Ab*HisG_S_: GenBank WP_001000724. *Ab*HisZ: GenBank WP_000155680. PDB ID: 8OY0

## Abbreviations

PRPP: 5-phospho-α-d-ribosyl-1-pyrophosphate
PRATP: *N*^1^-(5-phospho-β-d-ribosyl)-ATP
PP_i_: pyrophosphate
ATPPRT: ATP phosphoribosyltransferase
*Ab*HisG_S_: *A. baumannii* nonactivated HisG_S_
*Ab*ATPPRT: *A. baumannii* ATPPRT holoenzyme
*Ab*HisZ: *A. baumannii* HisZ
*k*_cat_: steady-state catalytic constant
DTT: dithiothreitol
MOPSO: 2-hydroxy-3-morpholinopropanesulfonic acid
AMPSO: *N*-(1,1-dimethyl-2-hydroxyethyl)-3-amino-2-hydroxypropanesulfonic acid
HEPES: 4-(2-hydroxyethyl)piperazine-1-ethanesulfonic acid,
PIPES: piperazine-*N,N*′-bis(2-ethanesulfonic acid)
CHES: *N*-cyclohexyl-2-aminoethanesulfonic acid
PEG-8000: polyethylene glycol 8000
*Mt*PPase: *Mycobacterium tuberculosis* pyrophosphatase
PRADP: *N*^1^-(5-phospho-β-d-ribosyl)-ADP
