# Supplementary material for: Crystal Structure, Steady-State, and Pre-Steady-State Kinetics of Acinetobacter baumannii ATP Phosphoribosyltransferase
Source: Biochemistry. 2023 Dec 27;63(2):230–40. doi: 10.1021/acs.biochem.3c00551 (PMC10795190; doi:10.1021/acs.biochem.3c00551)
Supplement: Supplementary file 1 — bi3c00551_si_001.pdf [file bi3c00551_si_001.pdf]

Supporting information

Crystal structure, steady-state and pre-steady-state kinetics of *Acinetobacter baumannii* ATP phosphoribosyltransferase

Benjamin J. Read,[†] Andrew F. Cadzow,[†] Magnus S. Alphey,[†] John B. O. Mitchell,[§] and Rafael G. da Silva[†,*]

[†]School of Biology, Biomedical Sciences Research Complex, University of St Andrews, St Andrews, KY16 9ST, United Kingdom

[§]EaStCHEM School of Chemistry, Biomedical Sciences Research Complex, University of St Andrews, St Andrews, KY16 9ST, United Kingdom

*To whom correspondence may be addressed: rgds@st-andrews.ac.uk, phone: +44 01334 463496

RESULTS

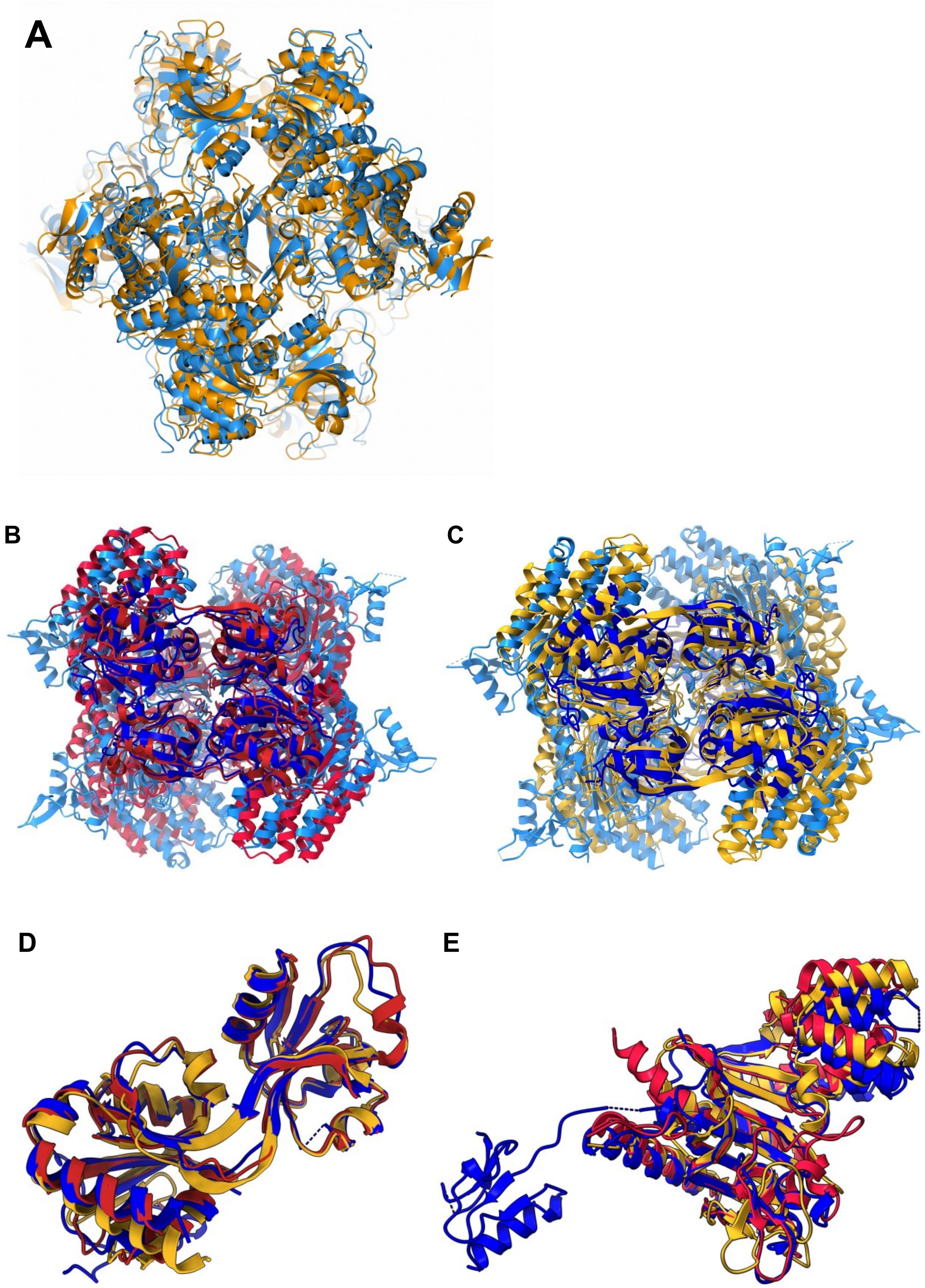

Figure S1. Ribbon representation of the overlaid *Ab*ATPPRT and the other short-form ATPPRT structures. (A) *Ab*ATPPRT (blue) and *P. arcticus* ATPPRT (5M8H) (gold). The RMSD between the two structures is 3.50 Å over 2113 Cα atoms. (B) *Ab*ATPPRT (*Ab*HisGs, dark blue; *Ab*HisZ, light blue) and *L. lactis* ATPPRT (1Z7M) (red). The RMSD between the two structures is 31.64 Å over 1973 Cα atoms. (C) *Ab*ATPPRT (*Ab*HisGs, dark blue; *Ab*HisZ, light blue) and *T. maritima* ATPPRT (1USY) (yellow). The RMSD between the two structures is 46.56 Å over 1833 Cα atoms. (D) *Ab*HisGs, *T. maritima* HisGs, and *L. lactis* HisGs (dark blue, yellow, red, respectively). The RMSDs between *Ab*HisGs and *T. maritima* HisGs, and *Ab*HisGs and *L. lactis* HisGs are 3.38 Å over 193 Cα atoms and 2.46 Å over 192 Cα atoms, respectively. (E) *Ab*HisZ, *T. maritima* HisZ, and *L. lactis* HisZ (dark blue, yellow, red, respectively). The RMSDs between *Ab*HisZ and *T. maritima* HisZ and *Ab*HisGs and *L. lactis* HisZ are 7.07 Å over 264 Cα atoms and 8.94 Å over 192 Cα atoms, respectively. Structures were overlaid, and RMSDs calculated, in ChimeraX.

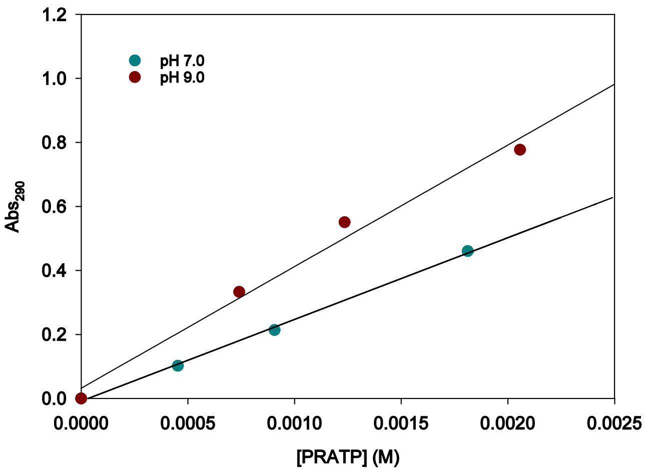

Figure S2. Determination of the molar extinction coefficient of PRATP at 290 nm at pHs 7.0 and 9.0. Data points are mean ± SD from three independent measurements. Lines represent linear regressions of the data. The path length for absorbance measurements was 0.1 cm.

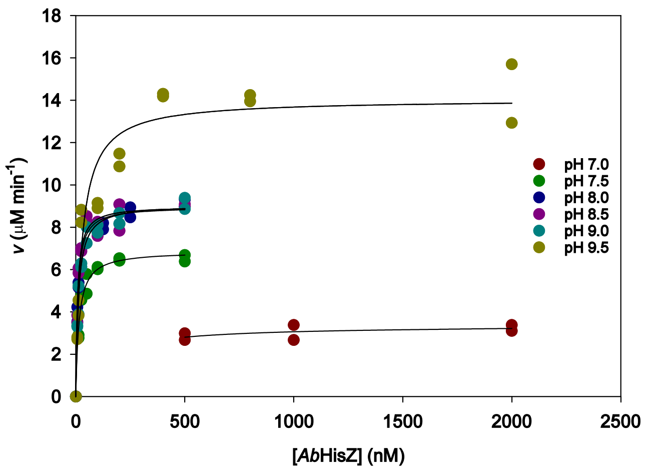

Figure S3. The pH-dependence of *Ab*HisG$_S$ allosteric activation by *Ab*HisZ. All data points for two independent measurements are shown. Lines are best fit of the data to eq 2.

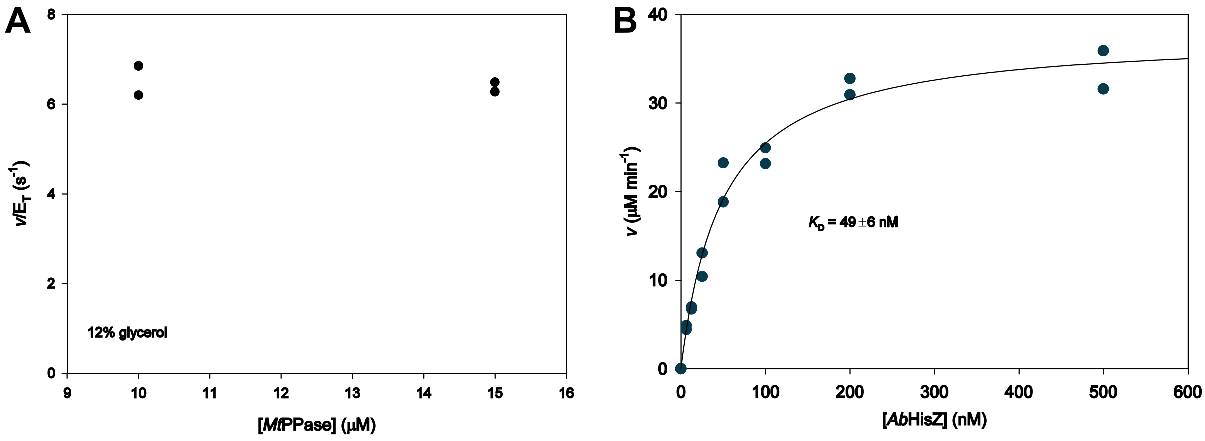

Figure S4. Effect of glycerol on *Ab*ATPPRT activity assay. (A) Effect of *Mt*PPase concentration on *Ab*ATPPRT rates in 12% glycerol. All data points for two independent measurements are shown. (B) Allosteric activation of *Ab*HisG$_S$ by *Ab*HisZ in 12% glycerol. All data points for two independent measurements are shown. Line is best fit to eq 1.

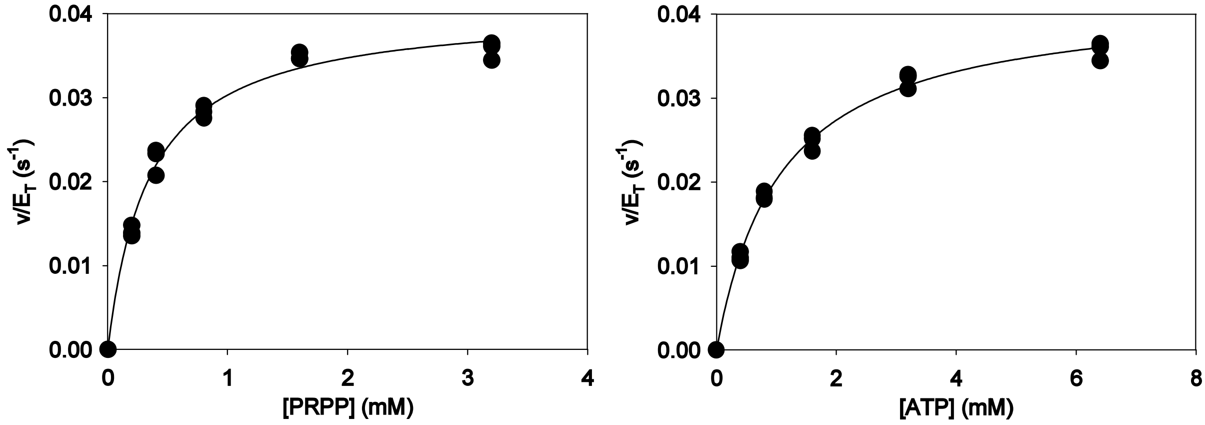

Figure S5. *Ab*HisGs substrate saturation curves at 5 °C. All data points for two independent measurements are shown. Lines are best fit to eq 3.

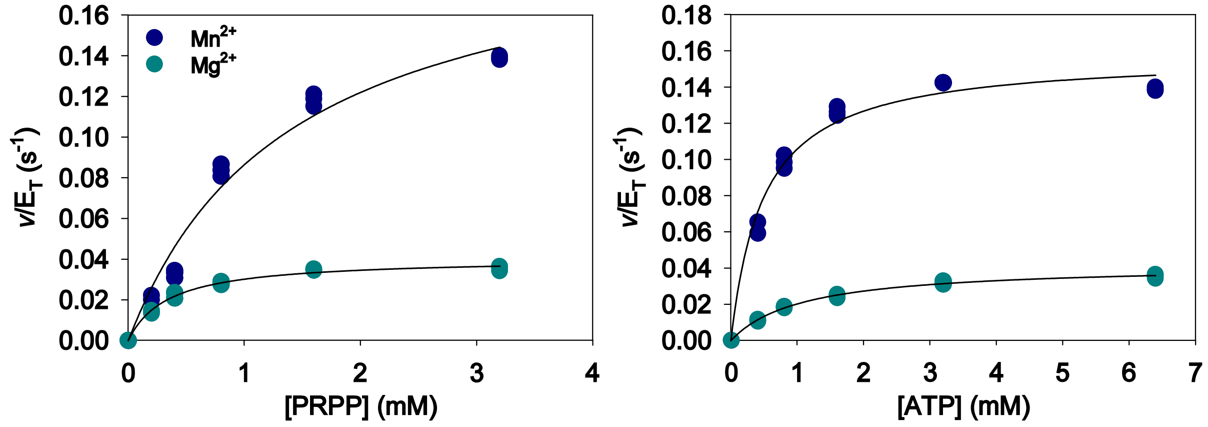

Figure S6. *Ab*HisGs steady-state kinetics with Mn^{2+} at 5 °C. All data points for three independent measurements are shown. Lines are best fit to eq 3.

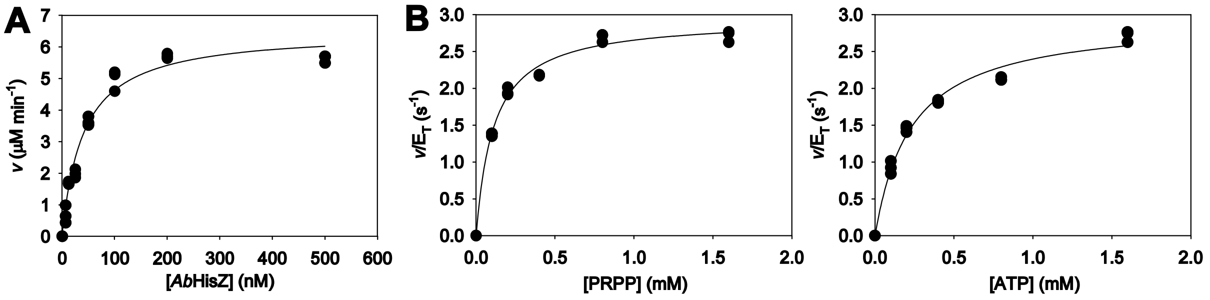

Figure S7. *Ab*ATPPRT steady-state kinetics at 5 °C. (A) *Ab*HisGs allosteric activation by *Ab*HisZ. All data points for three independent measurements are shown. Lines are best fit of the data to eq 2. (B) Substrate saturation curves. All data points for three independent measurements are shown. Lines are best fit to eq 3.

Table S1. Data processing and refinement statistics of *Ab*ATPPRT crystal structure.[a]

**Data Collection**	***Ab*ATPPRT**
**PDB ID**	8OY0
**Space group**	$P2_1$
**Cell dimensions**	
a, b, c **(Å)**	80.27, 172.82, 98.11
**α, β, γ (°)**	90.00, 90.97, 90.00
**Resolution (Å)**	30.82 – 2.40 (2.44 – 2.40)
R_{merge}	0.12 (1.05)
**I/σI**	9.6 (1.4)
**Completeness (%)**	95.6 (80.6)

**Redundancy**	6.4 (4.8)
**CC½**	0.996 (0.618)
**Refinement**	
**Resolution (Å)**	30.82 – 2.40
**No. reflections**	94223
**R_{work}/R_{free} (%)**	23.8/26.4
**Number of atoms**	
**Protein**	17980
**Ligand/ion**	6/4
**Water**	257
**B-factors**	
**Protein**	52.2
**Ligand/ion**	46.5/39.2
**Water**	35.7
**R.M.S. deviations**	
**Bond lengths (Å)**	0.004
**Bond angles (°)**	0.931
**Ramachandran**	
**Favoured (%)**	98

**Allowed (%)**	2
**Outliers (%)**	0

[a]Values in brackets are for the highest resolution shell.

Table S2. K_D values for *Ab*HisG$_S$-*Ab*HisZ interaction at different pHs. Each K_D is given as value ± fitting error to eq 2.

**pH**	K_D **(μM)**
**7.0**	<< 0.5[a]
**7.5**	0.014 ± 0.002
**8.0**	0.008 ± 0.001
**8.5**	0.007 ± 0.001
**9.0**	0.012 ± 0.001
**9.5**	0.027 ± 0.005

[a]Only an upper limit could be estimated.

Table S3. *Ab*HisG$_S$ and *Ab*ATPPRT steady-state kinetics at 5 °C.

**Enzyme**	**Metal**	k_{cat} **(s^{-1})**	K_M^{ATP} **(mM)**	K_M^{PRPP} **(mM)**	k_{cat}/K_M^{ATP} **(M^{-1} s^{-1})**	k_{cat}/K_M^{PRPP} **(M^{-1} s^{-1})**
***Ab*HisG$_S$**	Mg^{2+}	0.0413 ± 0.0006	1.07 ± 0.06	0.34 ± 0.03	39 ± 2	120 ± 10
	Mn^{2+}	0.18 ± 0.01	0.49 ± 0.06	1.4 ± 0.2	370 ± 50	130 ± 20
***Ab*ATPPRT**	Mg^{2+}	2.95 ± 0.05	0.23 ± 0.02	0.111 ± 0.008	13000 ± 1000	27000 ± 2000
